# Wearable and interactive multicolored photochromic fiber display

**DOI:** 10.1038/s41377-024-01383-8

**Published:** 2024-02-14

**Authors:** Pan Li, Yuwei Wang, Xiaoxian He, Yuyang Cui, Jingyu Ouyang, Ju Ouyang, Zicheng He, Jiayu Hu, Xiaojuan Liu, Hang Wei, Yu Wang, Xiaoling Lu, Qian Ji, Xinyuan Cai, Li Liu, Chong Hou, Ning Zhou, Shaowu Pan, Xiangru Wang, Huamin Zhou, Cheng-Wei Qiu, Yan-Qing Lu, Guangming Tao

**Affiliations:** 1 State Key Laboratory of Material Processing and Die & Mould Technology, School of Materials Science and Engineering and Wuhan National Laboratory for Optoelectronics, Huazhong University of Science and Technology, Wuhan, 430074 China; 2 Key Laboratory of Vascular Aging (HUST), Ministry of Education, Wuhan, 430030 China; 3 School of Optoelectronic Science and Engineering, University of Electronic Science and Technology of China, Chengdu, 611731 China; 4 Department of Electrical and Computer Engineering, National University of Singapore, Singapore, 117583 Singapore; 5 National Laboratory of Solid State Microstructures, Key Laboratory of Intelligent Optical Sensing and Manipulation, College of Engineering and Applied Sciences, and Collaborative Innovation Center of Advanced Microstructures, Nanjing University, Nanjing, 210023 China; 6 School of Performing Arts, Wuhan Conservatory of Music, Wuhan, 430060 China; 7 School of Mechanical Science and Engineering, Huazhong University of Science and Technology, Wuhan, 430074 China; 8 School of Architecture and Urban Planning, Huazhong University of Science and Technology, Wuhan, 430074 China; 9 School of Fashion, Beijing Institute of Fashion Technology, Beijing, 100029 China; 10 School of Optical and Electronic Information, Huazhong University of Science and Technology, Wuhan, 430074 China; 11 Tongji Medical College, Huazhong University of Science and Technology, Wuhan, 430074 China; 12 State Key Laboratory for Modification of Chemical Fibers and Polymer Materials, College of Materials Science and Engineering, Donghua University, Shanghai, 201620 China

**Keywords:** Photonic devices, Polymers, Displays, Fibre optics and optical communications, Optoelectronic devices and components

## Abstract

Endowing flexible and adaptable fiber devices with light-emitting capabilities has the potential to revolutionize the current design philosophy of intelligent, wearable interactive devices. However, significant challenges remain in developing fiber devices when it comes to achieving uniform and customizable light effects while utilizing lightweight hardware. Here, we introduce a mass-produced, wearable, and interactive photochromic fiber that provides uniform multicolored light control. We designed independent waveguides inside the fiber to maintain total internal reflection of light as it traverses the fiber. The impact of excessive light leakage on the overall illuminance can be reduced by utilizing the saturable absorption effect of fluorescent materials to ensure light emission uniformity along the transmission direction. In addition, we coupled various fluorescent composite materials inside the fiber to achieve artificially controllable spectral radiation of multiple color systems in a single fiber. We prepared fibers on mass-produced kilometer-long using the thermal drawing method. The fibers can be directly integrated into daily wearable devices or clothing in various patterns and combined with other signal input components to control and display patterns as needed. This work provides a new perspective and inspiration to the existing field of fiber display interaction, paving the way for future human–machine integration.

## Abstract (web summary)

We develop a wearable and interactive multicolored photochromic fiber using the thermal drawing technique, which overcomes the dependence on external light sources and the non-uniform light emission observed in polymer optical and photochromic fibers.

## Graphical abstract

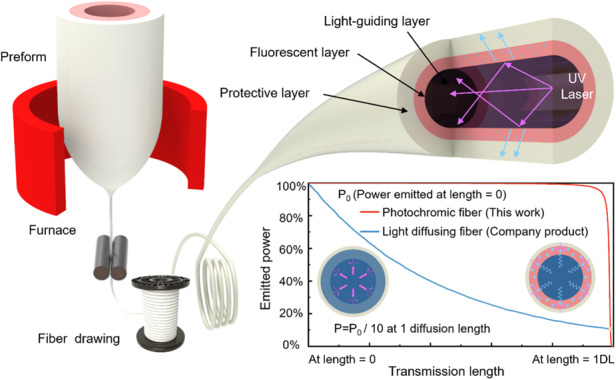

## Introduction

As material science advances, significant efforts have been made to develop functional fiber-based new materials and devices^1–9^. Smart fibers, when combined with existing mature textile engineering techniques, have the potential to meet user demands for softness, breathability, and comfort while being integrated directly into various textiles used in daily life^10^. Furthermore, information exchange is crucial in daily life. The efficiency and convenience of information transmission between humans and devices have stimulated the innovation of interactive devices^11–13^. As a means of interactive visualization in the smart textile field^14–16^, color-changing fibers break the traditional characteristics of hard and independent interaction interfaces^17^ and are expected to become an emerging interaction interface due to their excellent wearability and natural interactivity^18–21^.

Unlike traditional hard and flat interactive devices^4,22^, color-changing fibers offer good breathability and wear resistance. They can be twisted or bent in any direction^23–27^, making them adaptable to irregular body shapes. Additionally, they can be directly integrated into daily clothing using mature textile technology, serving as an “invisible” interaction interface^28–30^. There are two types of color-changing fibers: non-luminescent color-changing fibers and luminescent color-changing fibers. The former require external light sources to indicate an impending color change, limiting their applications to simple interaction or identification functions^18,31,32^. Achieving precise and controllable color changes with them is difficult. Conversely, luminescent color-changing fibers are capable of emitting light independently, displaying color changes in different wavelengths regardless of ambient brightness. This makes them ideal for display and interaction capabilities^33–37^. Examples of luminescent color-changing fibers include polymer optical fibers and light-diffusing fibers, which were initially used as line light sources for illumination devices^38^. The total internal reflection condition, which governs light propagation inside the fiber, was deliberately broken by introducing structural or material defects, thus actively inducing light leakage from the fiber^39^. However, due to transmission losses and artificial defects, the brightness uniformity of the fiber’s luminescence in the transmission direction and the leakage uniformity in the circumferential direction cannot be guaranteed, which significantly limits their application as line light sources^26,40,41^.

Inspired by photochromic fibers with fluorescence effects and polymer optical fibers that emit light when coupled with an external source, we present a wearable and interactive multicolored photochromic fiber using the thermal-drawing method to enhance the design versatility of fiber structures. We use polymethyl methacrylate material as the inner light-guiding layer and integrate fluorescent composite material with a lower refractive index in the outer layer. This coaxial structure allows for total internal reflection of light within the fiber while utilizing the wavelength conversion effect of the fluorescent material to achieve a uniform and comprehensive light emission. Furthermore, the fluorescent saturation effect mitigates the non-uniform effect of light absorption loss in the transmission direction. A variety of cross-sectional structures of the fiber were designed to expand the range of colors, allowing for the use of a single fiber in multiple color regulation and optimization of its color effect^42^. The fiber can be directly integrated into textile products of daily life due to its excellent wearability and programmability. Various luminous weaving patterns are displayed by controlling and adjusting the brightness of the specific light source at the end of the fiber. Furthermore, when combined with other sensing or image input elements^43^, it can achieve imperceptible, customizable, and expandable user display and interactive interfaces on pure textile products, paving the way for natural and interactive information display in daily textile products.

## Results

### Photochromic fiber design and fabrication

**Fig. 1:**
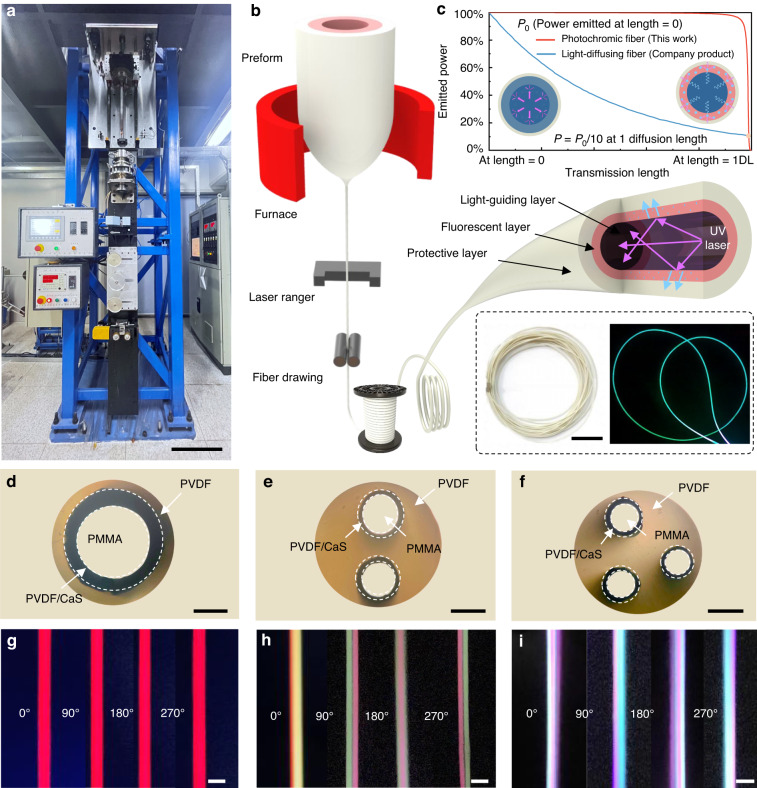
Fabrication and structural characterization of the photochromic fiber. **a** Photograph of the industrial-scale fabrication line of the photochromic fiber. The scale bar corresponds to 0.5 m. **b** Schematic illustration of the fabrication of the photochromic fiber. The inset shows a photograph of the fabricated and illuminated photochromic fiber. The scale bar corresponds to 10 cm. **c** Comparison of the luminescence attenuation in the transmission direction between this work and the commercial product (light-diffusing fiber)^[39]^. **d**–**f** Cross-sectional optical micrograph of three types of photochromic fiber, showing a different number of cores in the fiber, **d** for single-core red color, **e** for dual-core red and green colors, and **f** for tri-core red, green, and blue colors. The scale bar in each case corresponds to 200 μm. **g**–**i** Photographs of the photochromic fiber under radial observation, **g** for red color at 0°, 90°, 180°, and 270° angles, **h** for dual-core red and green colors at 0°, 90°, 180°, and 270° angles, and **i** for tri-core red, green, and blue colors at 0°, 90°, 180°, and 270° angles. The scale bar in each case corresponds to 500 μm

We used the thermal drawing technique for industrial-scale production of optical fibers. A micro-level preform structure was designed to control the internal microstructural characteristics of the fiber, allowing the production of kilometer-level photochromic fiber^44–46^. The preparation process is shown in Fig. 1a, b, where the preform is heated in a furnace until the bottom melts into a viscous state and then pulled down by gravity. The fiber diameters can be controlled from micrometers to millimeters by adjusting the fed and drawn speeds. The fabrication process of the preform is shown in Fig. S1 and described in the Methods section. The luminescence mechanism of the photochromic fiber is illustrated in Fig. 1b. When an external ultraviolet (UV) light source is coupled into a light-guiding layer made of Polymethyl methacrylate (PMMA), the UV light is guided by the waveguide effect within the light-guiding layer and undergoes total internal reflection transmission. As shown in Fig. S1a–d, a fluorescent layer composed of composites with polyvinylidene fluoride (PVDF) and inorganic phosphor particles (CaS) wrapped around the light-guiding layer are excited and emits visible light, which can be observed around the fiber. In addition, the fiber has an outer PVDF protective layer. In comparison to the commercial product (light-diffusing fiber), the photochromic fiber maintains good uniformity in the transmission direction due to the saturable absorption effect of fluorescent materials (Fig. 1c). The diffusion length (DL) is defined as the length where the emitted light power is 10% of the initial power by Corning® company. The high-quality shape retention of the entire fiber is demonstrated after the thermal drawing process (Fig. 1d). Several meters of photochromic fiber in different colors were fabricated (Fig. S1h–k).

We can integrate various fluorescent layers into the photochromic fiber to achieve multiple emission peaks that can be superimposed under different power ratios of coupled light sources. The overall light emission color in a single fiber can be controlled by adjusting these ratios, as demonstrated in typical dual-core and triple-core fiber structures (Fig. 1e, f). When excited by the coupled light source, these fibers can simultaneously emit various colors and exhibit uniform or multiple color distribution, which can be observed from different angles (Fig. 1g–i). These fibers have several advantages over other photoluminescent fibers. First, they can be directly coupled with an external light source and precisely controlled, eliminating the dependence on ambient light sources and significantly improving utilization efficiency. Second, the uniformity of the direction and circumferential radiation is ensured by encapsulating fluorescent materials within the core and controlling the internal microstructure of the fiber, resulting in a uniform overall visual light emission. Finally, the integral molding of the light-guiding and fluorescence layers significantly enhances the robustness and mechanical properties of the fibers.

### Luminescence performance of photochromic fiber

**Fig. 2:**
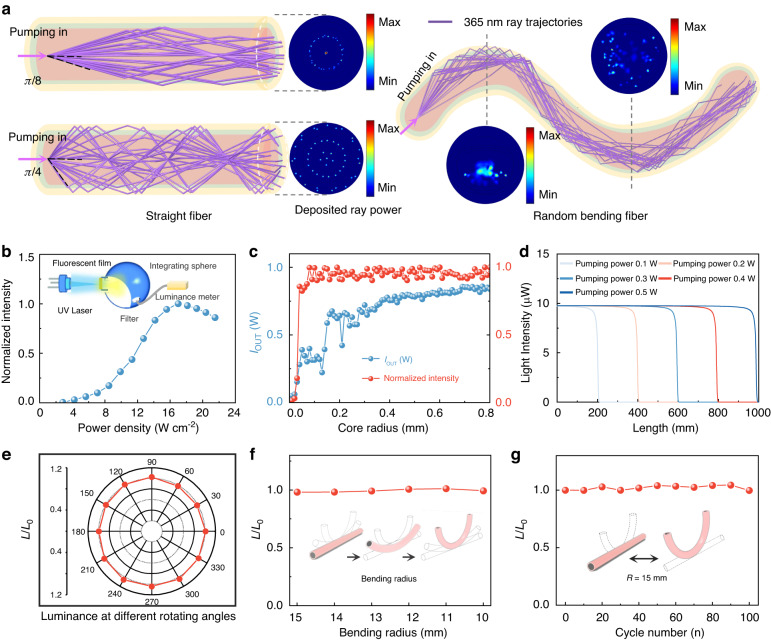
Luminescence performance of single-core photochromic fiber. **a** Ray model diagram of the single-core photochromic fiber, including straight or bending cases. The diagrams show the phenomenon of refraction and total internal reflection of light rays in the longitudinal section of the fiber. **b** Luminous saturation threshold of the single-core photochromic fiber. **c** Dependence of fluorescence intensity and pump intensity on core radius at the output port of a 3 mm length fiber. **d** Variation of fluorescence intensity with different pumping power in the direction of fiber length. **e** Luminance at different rotating angles mapped into polar coordinates, measured from 0° to 360° in 30° increments. **f** Dependence of luminance ratio on bending radius, the bending radius was varied from 15 mm to 10 mm. **g** Dependence of luminance ratio (*L*/*L*_0_) on bending cycle (radius of curvature *R* = 15 mm). *L*_0_ and *L* correspond to luminance before and after bending or torsion, respectively

A modeling investigation of the structural parameters and morphology characteristics of individual fibers was carried out. The material parameters were acquired from the experimental results (Fig. S2a, b). As shown in Fig. 2a, after the pumping ray beam is incident into the fiber, a total internal reflection occurs at the interface between the cladding and air, guiding the beam to transmit along the axial direction of the fiber. The power of pumping light in the radial direction of the fiber can be obtained through simulation. the quantum yield of fluorescence material refers to the ratio of the number of photons emitting fluorescence to the number of photons absorbed. According to this parameter, the luminous intensity of the material can be calculated. As shown in Fig. 2b, the quantum yields (~11.8%) of the fluorescent material and its saturated absorption threshold for the pumping light are experimentally measured. These two parameters are used as input data for the light transmission loss simulation in the axial direction of the fiber. The fiber core size is the parameter with the greatest influence on the light transmission loss. As shown in Fig. 2c, when the fiber length is fixed, the energy of pumping light and emitted fluorescence light at the fiber port show an overall upward trend with increased fiber core size, eventually reaching a plateau. Therefore, the core radius is set to 400 μm in the following experiment. Based on the saturated absorption effect of photochromic light-emitting material, its absorption coefficient can be written as ${\alpha }{=}{{\alpha }}_{{0}}{/}{(}{1}{+}{I}{/}{{I}}_{{s}}{)}$, where ${{\alpha }}_{{0}}$ is the absorption coefficient of the material for low pumping energy, ${I}$ is the intensity of pumping light, and ${{I}}_{{s}}$ is the saturation threshold intensity. The formula shows that when the pumping energy is much larger than ${{I}}_{{s}}$, the luminous intensity of the luminescent material is not affected and maintains a uniform luminous intensity. The luminescence loss performance along the axial direction is calculated for single-end pumping. The luminescence remains uniform in the transmission direction until the saturation threshold is reached. As shown in Fig. 2d, when the pumping intensity is 0.5 W, the luminescence remains uniform for 1 m. If the fiber is double-ended pumped, it will transmit at least a 2 m distance with the same luminosity.

Due to its unique fiber-shaped morphology, the photochromic fiber can emit light in any direction around the circumference as a linear light source. The relative brightness distribution of the fiber measured around its circumference is shown in Fig. 2e, which indicates that the intensity along the circumference at different locations was almost identical. Regarding its mechanical properties, the excellent flexibility of the fiber ensures its good weaving performance. To verify the flexibility and the effect of fiber bending on the internal light-guiding loss performance, we investigated the relationship between different degrees of fiber bending and the emission intensity. When the bending radius was decreased from 15 mm to 10 mm, the brightness variation was less than 1% (Fig. 2f), indicating that the fiber could retain its luminous performance when woven into textiles. The fatigue properties of the fiber were then tested. After 100 times bending with a radius of curvature of 15 mm, its brightness was maintained at above 98% of its maximum value (Fig. 2g), indicating that the woven pattern of fiber could meet the flexibility requirements of daily wear. In addition, the fiber could withstand a maximum stress of 65 N/mm^2^, with a maximum strain of 7%, and good stiffness (Fig. S2c). The gray-scale grade of the color fastness to water (GB/T 5713-2013) and rubbing crocking (GB/T 3920-2008) was 4–5, and the Martindale abrasion resistance showed no damage after 25,600 cycles under a loading of 12 kPa (GB/T 21196.4-2007), demonstrating its suitability for daily use. We also tested the fiber for color fastness to light and heat. According to the test method of GB/T 8427-2019, the gray-scale grade of the color fastness to light and heat was 4–5, which meant that the fiber’s color change and color stain were negligible or no change after light 192 h in UV irradiation with 42 W/m^2^@300–400 nm. Based on the trichromatic theory, three types of fibers were prepared. Each fiber was tested for fluorescence spectrum emission using a 365 nm UV light source. The results showed that the emission peaks of red, green, and blue photochromic fiber were at 654 nm, 515 nm, and 451 nm, respectively (Fig. S2d). According to the CIE 1931 standard, the CIE coordinates were (0.186, 0.128), (0.496, 0.304), and (0.217, 0.507), representing the typical blue, red, and green colors, respectively. In addition, the fibers could exhibit good luminescent performance in extreme environments such as 100 °C boiling waters (Fig. S3a, and Supplementary Video 1), strong acid (Fig. S3b, and Supplementary Video 2), and strong alkali (Fig. S3c, and Supplementary Video 3).

**Fig. 3:**
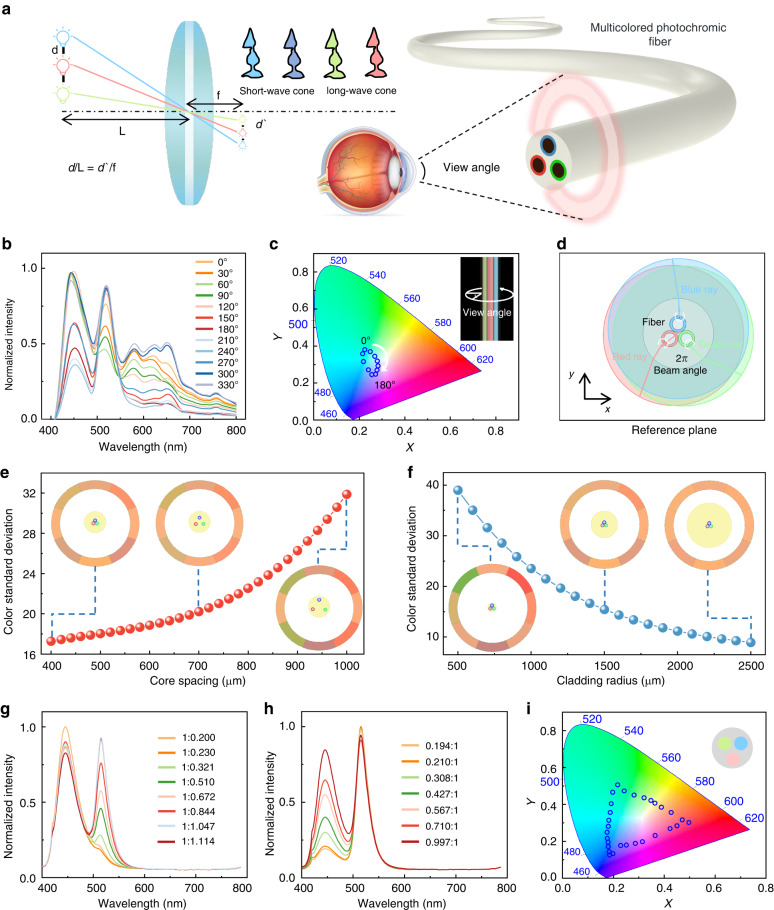
Luminescence performance of multicolored photochromic fiber. **a** Schematic illustration of multicore photochromic fiber as viewed from the human eye. Adjusting the fiber shape and size parameters allows the circumferential color difference and fiber multi-color mixing effect to be optimized, and the discernible visual distance can be minimized. **b** The circumferential spectra of multicolored photochromic fiber, measured from 0° to 360° at 30° increments. **c** Dependence of *x*, *y* chromaticity coordinate on viewing angle. **d** Schematic diagram of the light field distribution radiation of three-color fluorescence in the fiber cross-section. **e** Dependence of color standard deviation on core spacing. **f** Dependence of color standard deviation on cladding radius. **g** Luminescence spectrum with the brightness ratios of blue to green shown on the right. The power of the blue-core coupling light source is unchanged, while the power of the green-core coupling light source is adjusted. **h** Luminescence spectrum with the brightness ratios of green to blue shown on the right. The power of the green-core coupling light source is unchanged, while the power of the blue-core coupling light source is adjusted. **i** *x*, *y* chromaticity coordinates are controlled by adjusting the light power of different cores. The chromaticity triangle is composed of the power ratios of the light sources coupled to the red, green, and blue cores, with the coordinates of the vertices from top to bottom being (0.217, 0.507), (0.496, 0.304), and (0.186, 0.128), respectively. The fiber can achieve all chromaticity values within the triangle by mixing light sources with different power ratios

As previously mentioned, emitting different colors in a single fiber remains a challenge. Here, we used thermal drawing methods to regulate the inner structure of the fiber by encapsulating multiple light-guiding core layers with various colored fluorescent layers in a single fiber. We successfully modulated the emitted spectra within a specific range by adjusting the power of the light sources coupled with the different cores. We can not only achieve multi-color control in a single fiber but also arbitrarily adjust the shape and structural parameters of the preform by thermal drawing technique. We can minimize the chromatic aberration observed from different viewpoints by optimizing the size of the multicore fiber cladding and the distance between the light-guiding cores so that the colors appear as consistent as possible. The human eye is regarded as a lens to describe the critical distance *L* of the color mixing of multicore fiber. The critical distance *L*, as defined by the lens imaging principle, is the distance between the image points of the three fluorescent sources after the eye imaging just on the same cone cell. Cone cells are responsible for detecting color in our vision. The schematic diagram of the model is shown in Fig. 3a. Human eye’s resolution to multicolor is determined by the distance between the luminescence centers, i.e., the edge distance between the centers of light-guiding cores. The distance between the cores is set to 400 μm (The multicore with 3 μm edge distance), and the critical distance *L* is ~1 mm.

Moreover, because the multicore fiber contains multiple light sources, we can optimize the distance between the light-guiding cores to maintain good color mixing at observation distance. The spectral radiation intensity varies when different directions are examined due to the unique asymmetrical internal structure of a multicore fiber. Therefore, we measured the excitation spectra at different angles while driving the coupled light source (Fig. 3b, Fig. S4a) and plotted the direction chromaticity coordinates on a chromaticity diagram (Fig. 3c, Fig. S4b). The excitation spectra across the cylindrical surface showed cyclic variation. The light field distribution radiation of three-color fluorescence in the fiber cross-section was modeled using numerical finite element methods in Fig. 3d. The ray power of each fluorescent source from eight viewpoints is obtained by the commercial software Multiphysics COMSOL. Simulation shows that the chromatic aberration of the multicore fiber increases as the core spacing increases (Fig. 3e). In addition, the chromatic aberration is also related to the radius of the multicore fiber cladding. According to the relationship between the circular cladding radius and the color standard deviation in Fig. 3f, the optimal cladding radius was 1500 μm for better mechanical properties, and the optimized color mixing results were shown in the inset.

In addition to changing the observation angle of multicore fibers to regulate a broader color spectrum, we can also exploit the segmented control of the light-guiding cores, which allows for independent regulation of their luminance and, hence, a broader color gamut. As a demonstration, we designed a tri-core fiber with three light-guiding cores for red, green, and blue. By adjusting the luminance of each light-guiding core independently through the coupling of different power sources at their ends, we achieved different mixing chromaticity effects within the human eye’s perception at a macro level. Specifically, while maintaining a constant power input for the blue light-guiding core, we varied the power input for the green light-guiding core, increasing the luminance ratio from 0.200 to 1.114 and adjusting the excitation spectrum (Fig. 3g). We subsequently kept the power input of the green light-guiding core constant and varied the power input of the blue light-guiding core, increasing the luminance ratio from 0.194 to 0.997 and adjusting the excitation spectrum (Fig. 3h), changed the *x*, *y* chromaticity coordinates were from (0.182, 0.122) to (0.198, 0.467) (Fig. S4c). This segmentation approach to coupling light-guiding cores can meet the demand for a single fiber that can facilitate multicolored adjustable applications. Similarly, we coupled the light sources of the red, green, and blue light-guiding cores in pairs with different power ratios (Fig. S4d–i) and then plotted the resulting color range of the coordinates on a color gamut chart (Fig. 3i).

### Application scenarios of photochromic fiber

**Fig. 4:**
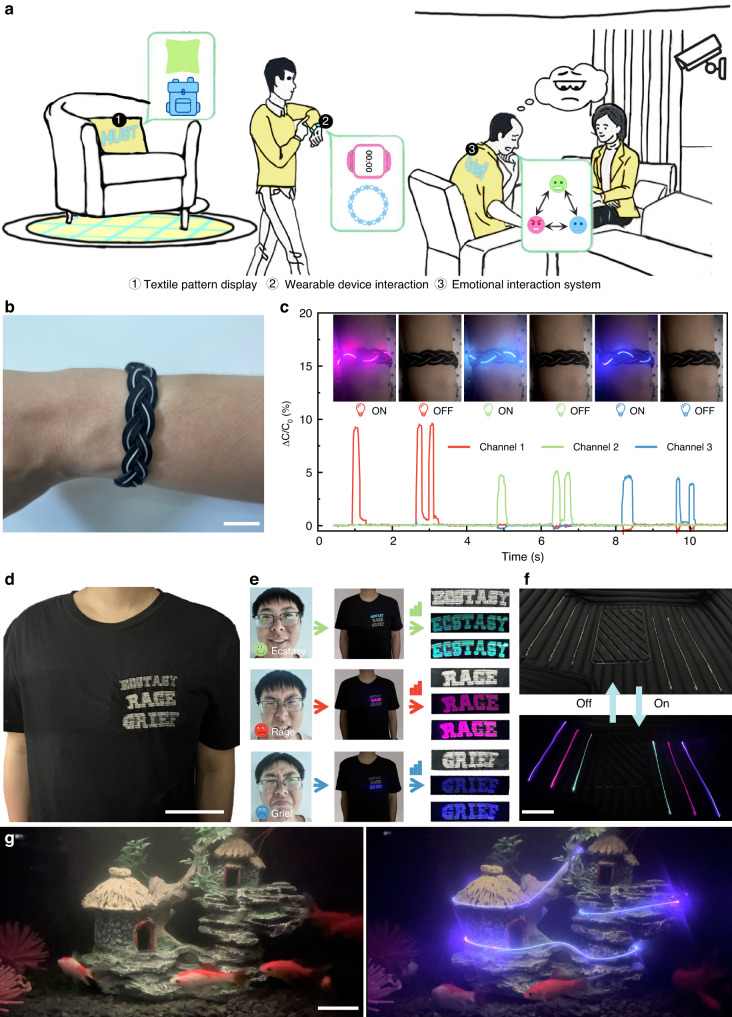
Application scenarios of the photochromic fiber systems. **a** Schematic illustration for the interaction system based on photochromic fiber. **b** Photograph of the wearable wristband. The scale bar corresponds to 2 cm. **c** Correspondence between capacitance response and light-emitting colors under different touch positions. **d** Photograph of the photochromic fiber integrated into T-shirts. The scale bar corresponds to a 10 cm. **e** Wearable interactive display system that reflects the user’s current emotional state based on his facial expression. **f** Photograph of the photochromic fiber in automotive interiors. The scale bar corresponds to 10 cm. **g** Photochromic fiber arranged in a fish tank to demonstrate its underwater illumination. The scale bar corresponds to 5 cm

In comparison to light-emitting fibers with another mechanism that requires complex energy storage devices and driving hardware, the photochromic fibers we developed have superior performance and are more suitable for a variety of small and lightweight wearable devices (Table S1). We can maintain the overall display effect of the fiber pattern by controlling the switch and brightness of the light source coupled with each fiber segment, thereby enriching the display content of the fabric itself. To emphasize their potential as a natural interface for human–machine interaction, we have incorporated photochromic fiber in daily textile products (Fig. 4a). We used embroidery to sew various letter patterns on the cross-stitch or cotton fabrics, using various colors of photochromic fiber to demonstrate the designability and wearable of luminous patterns (Fig. S5a). We wove the capacitance sensing yarn that is stable in existing work performance with the photochromic fiber into daily textiles^47^, and designed an integrated wearable textile product (A wearable wristband) that integrates perception interaction and luminous display (Fig. 4b, and Fig. S5b, c). By making different hand gestures, capacitance changes in each channel can be used to control the on-and-off state of three different colors of the fibers on the wristband (Fig. 4c, and Supplementary Video 4). In addition, we designed a musical metronome with this wristband that dynamically interacts with the music as it is accompanied in real-time (Supplementary Video 5). This helps musicians keep up with the rhythm through visual stimuli, thereby avoiding difficulties in hearing the beat in noisy environments.

To demonstrate the potential of fabric displays in human social interaction, we utilized photochromic fiber to create a fabric display screen that can reflect the user’s emotions in real time based on their facial expressions. We created an 87 mm × 76 mm textile pattern on a cotton T-shirt by sewing three types of single-core photochromic fiber representing joyful, angry, and sad emotional states to form letter patterns (Fig. 4d, and Fig. S5d, e). It is simple to control the color displays in each specified way using a designed circuit. The patterns with varying brightness can be accurately displayed by manipulating the light source coupled with the fibers. We have combined our developed facial expression detection algorithm with displayable textiles. It classifies emotions such as joy, anger, and sadness with the highest confidence score^48^, using an external camera that feeds the algorithm’s current facial expressions, serving as a referential for evaluating the intensity of the displayed emotion. This emotion analysis result and confidence score are then communicated to the lower-level machine control board, which drives the light source to illuminate the corresponding representative patterns on the fabric and adjusts the brightness based on the intensity of the displayed emotion. We set the image-capturing cycle of the camera and the data-sending cycle of the control board to ten seconds. This means that the entire emotion display system can classify and analyze the user’s current emotions and drive the corresponding implementation of patterns stitched onto the fabric within ten seconds (Fig. 4e and Supplementary Videos 6 and 7). This provides a new solution to interpersonal emotional interaction and communication. In the future, with the emergence of better emotion decoding methods, we believe that the luminous textile we developed could become a useful communication aid. In addition, we applied the fibers to automotive interiors to demonstrate their visual interaction characteristics. The fibers arranged on the car’s cushions (Fig. 4f and Fig. S5f) can be controlled to emit various color lights as required (Supplementary Video 8), which can also be combined with in-car music to realize dynamic rhythms. In order to demonstrate its underwater lighting effect, the fibers were arranged in a fish tank, which can still illuminate after a long immersion (Fig. 4g, and Supplementary Video 9). This will be helpful for applications in deep-sea rescue, undersea exploration, and offshore fishing.

## Discussion

To summarize, we have developed a mass-producible photochromic fiber using the thermal drawing technique. The developed fibers overcame the dependence on external light sources and the non-uniform light emission observed in traditional polymer optical and photochromic fibers. By utilizing internal structural modulation techniques, the total internal reflection was maintained while inducing the light leakage caused by the wavelength conversion effect of fluorescent materials. The saturable absorption effects were employed to mitigate the negative impact of excessive light leakage on fiber luminosity, ensuring uniform luminescence along the fiber. Moreover, multicolor display in a single fiber was achieved by regulating the fiber structure and optimizing mixed-color effects through design and structural analysis. The fibers were combined with other perceptual interaction components and computer terminals to enable dynamic information exchange through external signal control of fiber pattern and brightness. This work provides new scenarios for the interactive application of wearable luminous devices in daily life.

## Materials and methods

### Preparation of the fluorescent composites

To prepare the fluorescent composites, the base temperature of the micro twin-screw extrusion device was maintained at 160 °C (first zone), 180 °C (second zone), and 185 °C (third zone). Inorganic fluorescent powder (Guangdong Huanasi Industrial Co., Ltd.) and polyvinylidene fluoride particles (Jiangxi Dasheng Plastic Fiber Co., Ltd.) were fed into the two hoppers of the micro twin-screw extrusion device (Model SJZS-10B, Wuhan Ruiming Experimental Instrument Co., Ltd.) in a mass ratio of 1:5.669. The handle was rotated following the device’s direction, allowing for even heating inside the device. The device started, and the main machine speed was adjusted manually. The charging area was set to automatic, and the adjustments were made automatically. After running the device for some time, the composite material was extruded through the outlet die and transferred to the air-cooling device for cooling. The extruded material was then directly fed into the plastic pelletizer for pelletizing. A thoroughly mixed and uniformly distributed fluorescent composite particle was obtained after repeating the cycle several times.

### Preparation of the fluorescent composite film

The fluorescent composites film was prepared by putting the fluorescent composites material into the hot-pressing mold, setting the temperature of both the upper and lower die of the hot-press to 160 °C, and pre-pressing the composites particles under the pressure of 0.3 MPa for 1 min to soften the composite particles. The pressure was gradually increased to 2 MPa, 5 MPa, 15 MPa, and 25 MPa, with an interval of about 30 s. After pressurizing up to 25 MPa and holding pressure for around 15 min, the composite film was obtained with a thickness of 200 μm.

### Preparation of polyvinylidene fluoride film

A polyvinylidene fluoride film with a thickness of 200 μm was prepared by hot-pressing the polyvinylidene fluoride particles according to the same method mentioned above (Preparation of the fluorescent composites film).

### Preparation of the waveguide preform

The modified PMMA particles (Jiangxi Dasheng Plastic Optical Fiber Co., Ltd.) were put into the hot-pressing mold with a stainless steel groove measuring 100 mm in length, 10 mm in width, and 10 mm in height. Both sides of the model were covered with steel plates to ensure the materials’ uniform pressure during the hot-pressing process. The temperature of the upper and lower die was set to 180 °C. The modified PMMA particles were preheated under the pressure of 0.3 MPa for 3 min. The pressure was then increased to 2.5 MPa for 15 min to compact the material in the mold. These steps were repeated several times until the waveguide preform was formed. Finally, the preform was processed on a lathe and turned into a rod with a 10 mm diameter.

### Preparation of the photochromic fiber preform

The prepared fluorescent composite film and polyvinylidene fluoride film were cut into rectangles with a width equivalent to the length of the waveguide preform. Then, the fluorescent composite film was wound on the waveguide preform, which was thermally cured in a muffle furnace at a temperature of 190 °C for 30 min. During this process, the preform was continuously tumbled to enable the uniform thermal consolidation of the composite film. These steps were repeated several times until the diameter of the preform was greater than 13 mm, and then the preform was processed on a lathe and turned into a rod with a 12.5 mm diameter. Thus, the fluorescent composites preform were obtained with a diameter of 12.5 mm. Subsequently, the polyvinylidene fluoride film was wound on the fluorescent composites preform and thermally cured in a muffle furnace at a temperature of 190 °C for 60 min. During this process, the preform was continuously rolled to uniformly achieve the film’s overall thermal-curing. These steps were repeated several times until the diameter of the preform was greater than 16 mm, and then the preform was processed on a lathe and turned into a rod with a 15 mm diameter. Finally, the photochromic fiber preform with a diameter of 15 mm was obtained. The thickness of the fluorescent layer (i.e., the fluorescent composites film wound on the preform) was 2.5 mm, and the thickness of the outer cladding layer (i.e., the polyvinylidene fluoride film wound on the preform) was 2.5 mm.

### Preparation of multicolored photochromic fiber preform

Two steps were required to prepare the multicolored photochromic fiber preform: (1) preparation of the fluorescent composites and film. Two or three different fluorescent composite films with a thickness of 200 μm can be prepared using a similar method mentioned above (Preparation of the fluorescent composite film). (2) Preparation of multicolored photochromic fiber preform. First, prepare the waveguide preform. The modified PMMA particles were put into the hot-pressing mold with a stainless steel groove measuring 100 mm in length, 5 mm in width, and 5 mm in height. Both sides of the model were covered with steel plates to ensure the materials’ uniform pressure during the hot-pressing process. The temperature of the upper and lower die was set to 180 °C. The modified PMMA particles were preheated under the pressure of 0.3 MPa for 3 min. The pressure was then increased to 2.5 MPa for 15 min to compact the material in the mold. These steps were repeated several times until the waveguide preform was formed. The preform was processed on a lathe and turned into a rod with a 4 mm diameter. Two or three waveguides preform with a 4 mm diameter can be prepared using a similar method. Second. the fluorescent composite film was wound on the waveguide preform to form a coiled preform with a diameter of 5 mm. Two or three of these coiled preforms were prepared using this method. Then, prepare the polyvinylidene fluoride preform. The polyvinylidene fluoride particles were put into a hot-press mold with a stainless steel groove measuring 100 mm in length, 16 mm in width, and 16 mm in height., and turned into a cylindrical preform with a diameter of 16 mm using a lathe. The exact number of through-holes as the coiled preforms (Two or three) were drilled in the vertical axis of the cylindrical preform. Finally, the coiled preforms were inserted into multiple 5 mm holes and heat-set again to obtain the multicolored photochromic fiber preform.

### Thermal drawing of photochromic fiber preform

To thermal-draw the photochromic fiber preforms, a 1 mm diameter hole was drilled along the radial direction at 2 mm from the bottom of the preform. A stainless steel wire was bound to hang 10 g weights through the radial hole. The temperature of the furnace was set to 200 °C and 280 °C, respectively. The fibers were then moved to the traction device after the material head was dropped. The feeding speed was set to 0.2 mm/min, and the drawing speed was 0.16 m/min. Finally, the photochromic fiber with a uniform and stable diameter of about 600 μm was drawn.

### Thermal drawing of the multicolored photochromic fiber preform

The temperature of the furnace was set to 200 °C and 280 °C, respectively. The fibers were then moved to the traction device after the material head was dropped. The feeding speed was set to 0.2 mm/min, and the multicolored photochromic fiber with a uniform and stable diameter was drawn.

### Structure and performance characterization

The cross-section of the photochromic fiber was characterized by an optical microscope (CX40M, Shunyu Optical Technology Co., Ltd.). The saturation threshold test of the fluorescent composite material was conducted using a brightness meter (Asteria, ADMESY), a filter under the working conditions of a solid-state laser source (MBL-FN-473-500mW, Changchun Xinchuang Industry and Technology Co., Ltd.), and an integrating sphere in the visible light range (K SPHERE-Petit e, Horiba). A semiconductor laser with a wavelength of 365 nm was used to characterize the photochromic fiber as single-core, dual-core, or tri-core. The radius and shape of the laser spot could be controlled through the spot shaping mirrors at the output of the laser, and then the adjusted spot is coupled to the waveguide core of the photochromic fiber^49–51^. The brightness test of the photochromic fiber was measured using a brightness meter (Asteria, ADMESY). An ultraviolet light source (NDV4312, NICHIA) was used for light coupling in wearable scenarios (The working area is only about 3.5 mm in diameter, and the largest size of the contour diameter is 5.6 mm). The circumferential performance test was conducted using a turntable (RS60-L, Jiangxi Fala Automation Technology Co., Ltd.). The spectrum of the photochromic fiber was measured using a spectrophotometer (MS2000, OCEANHOOD). The actual photos in this article were taken using the original camera.

### Simulation

Simulations were performed using the geometrical optics module of the COMSOL Multiphysics 5.6. The three-dimensional simulation model of single-core photochromic fiber was three coaxial cylinders with 3 mm length. The three domains are the core layer, fluorescent composite film, and cladding layer from inside to outside, and the corresponding real parts of the refractive index are 1.48, 1.44, and 1.42, respectively. The absorption of fluorescent composite film to pumping light is included in the imaginary part of the refractive index. The relationship between the absorption coefficient ${{\rm{\alpha }}}$ of the fluorescent composite film and the imaginary part ${n}_{i}$ of its refractive index is ${\rm{\alpha }}{=}{4}{{\rm{\pi }}}{{n}}_{{i}}{/}{{\rm{\alpha }}}{=}4{\rm{\pi }}{n}_{i}/\lambda$. These material parameters were acquired from the experimental results. A frozen wall was set at the end face of the fiber to calculate the deposition ray power. The simulation of the circumferential luminescence uniformity of multi-core fibers was based on the two-dimensional model of the fiber cross-section. Every annular fluorescence emission area has a spherical ray direction vector with different vacuum wavelengths. Eight frozen walls were set at 5 mm from the cladding center to simulate eight views in the circumferential direction. The deposited ray power on these walls was calculated for each fluorescence, and the mixing colors in different views were obtained.

### Supplementary information

Supplementary information

Video_Overview

Supplementary Video 1

Supplementary Video 2

Supplementary Video 3

Supplementary Video 4

Supplementary Video 5

Supplementary Video 6

Supplementary Video 7

Supplementary Video 8

Supplementary Video 9

## Data Availability

All data are available in the main text or the supplementary materials. Information requests should be directed to the corresponding authors.
