# Supplementary material for: Wearable and interactive multicolored photochromic fiber display
Source: Light Sci Appl. 2024 Feb 14;13:48. doi: 10.1038/s41377-024-01383-8 (PMC10866970; doi:10.1038/s41377-024-01383-8)
Supplement: Supplementary file 1 — Supplementary information [file 41377_2024_1383_MOESM1_ESM.docx]

Supplementary Information for

**Wearable and Interactive Multicolored Photochromic Fiber Display**

Pan Li^1, 2^†, Yuwei Wang^1, 2^†, Xiaoxian He^3^†, Yuyang Cui^1, 2^, Jingyu Ouyang^1, 2^, Ju Ouyang^1, 2^, Zicheng He^1, 2^, Jiayu Hu^1, 2^, Xiaojuan Liu^1, 2^, Hang Wei^4^, Yu Wang^5^, Xiaoling Lu^6^, Qian Ji^7^, Xinyuan Cai^8^, Li Liu^9^, Chong Hou^1, 2, 10^, Ning Zhou^2, 11^, Shaowu Pan^12^, Xiangru Wang^3^, Huamin Zhou^1^, Cheng-Wei Qiu^4^, Yan-Qing Lu^5^*, Guangming Tao^1, 2^*

1. State Key Laboratory of Material Processing and Die & Mould Technology, School of Materials Science and Engineering and Wuhan National Laboratory for Optoelectronics, Huazhong University of Science and Technology, Wuhan 430074, China

2. Key Laboratory of Vascular Aging (HUST), Ministry of Education, Wuhan 430030, China

3. School of Optoelectronic Science and Engineering, University of Electronic Science and Technology of China, Chengdu 611731, China

4. Department of Electrical and Computer Engineering, National University of Singapore, Singapore 117583, Singapore

5. National Laboratory of Solid State Microstructures, Key Laboratory of Intelligent Optical Sensing and Manipulation, College of Engineering and Applied Sciences, and Collaborative Innovation Center of Advanced Microstructures, Nanjing University, Nanjing, 210023, China

6. School of Performing Arts, Wuhan Conservatory of Music, Wuhan 430060, China

7. School of Mechanical Science and Engineering, Huazhong University of Science and Technology, Wuhan 430074, China

8. School of Architecture and Urban Planning, Huazhong University of Science and Technology, Wuhan 430074, China

9. School of Fashion, Beijing Institute of Fashion Technology, Beijing, 100029, China

10. School of Optical and Electronic Information, Huazhong University of Science and Technology, Wuhan 430074, China

11 Tongji Medical College, Huazhong University of Science and Technology; Wuhan 430074, China

12. State Key Laboratory for Modification of Chemical Fibers and Polymer Materials, College of Materials Science and Engineering, Donghua University, Shanghai 201620, China

†These authors contributed equally to this work.

*Corresponding author. Email:

[tao@hust.edu.cn, yqlu@nju.edu.cn](mailto:tao@hust.edu.cn, )

Supplementary Figures


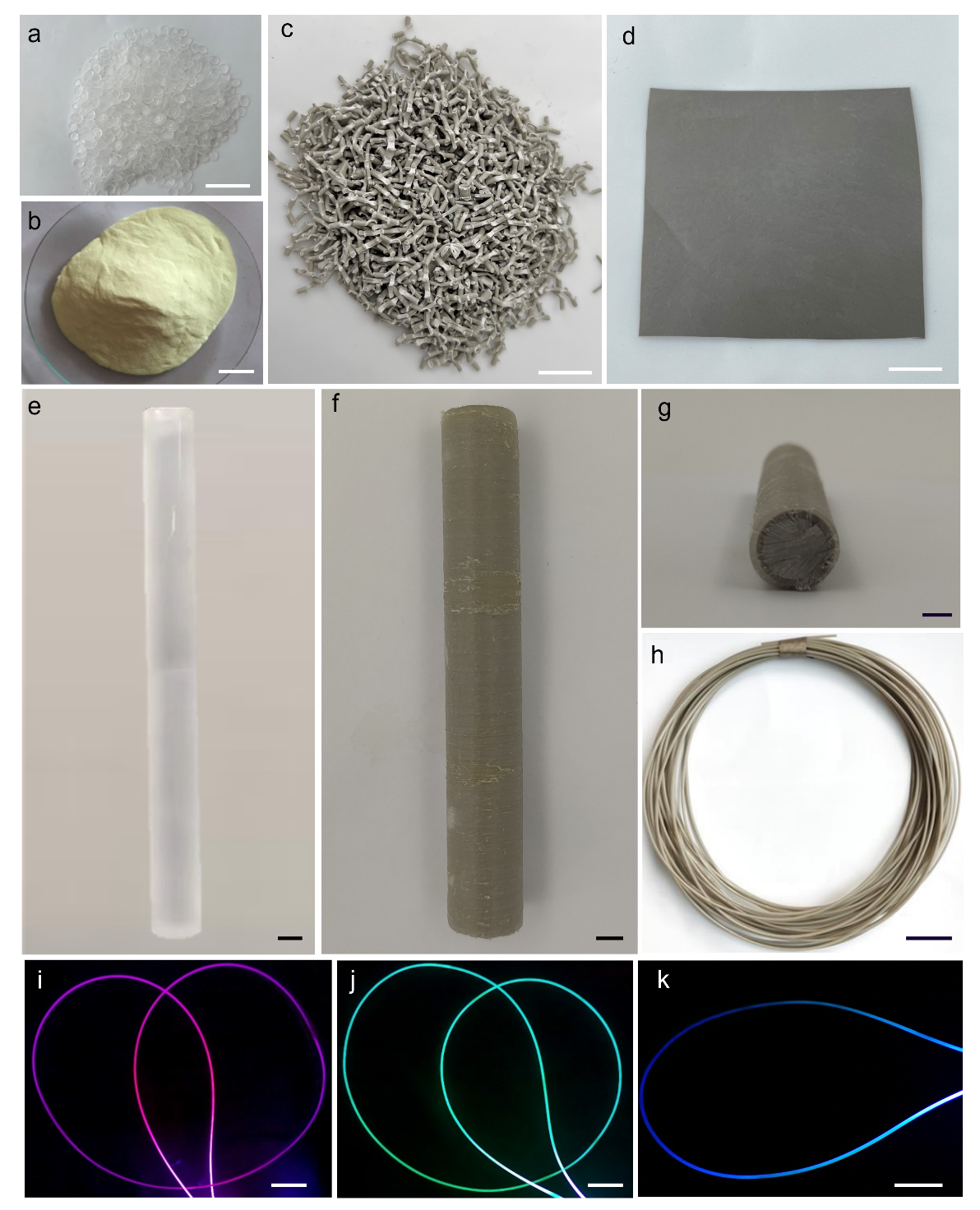


**Fig. S1 | Fabrication of photochromic fiber preform and photograph of the thermal drawing fiber.** **a** Photograph of PVDF particles. Scale bar, 2 cm. **b** Photograph of inorganic phosphor particles. Scale bar, 1 cm. **c** Fluorescent composites, obtained by melt blending of PVDF particles and inorganic phosphor particles. Scale bar, 5 cm. **d** Fluorescent composite film, obtained by hot pressing. Scale bar, 5 cm. **e** Photograph of the light-guiding core preform. Scale bar, 5 cm. **f-g** Photograph of the photochromic fiber preform. Scale bar, 5 cm. **h** Photograph of the photochromic fiber, obtained by thermal-drawing. Scale bar, 5 cm. **i-k** Red/ blue/green color of photochromic fiber. Due to the double-ended pump in low power, the brightness at the port of the photochromic fiber is brighter than in the middle region. Scale bar, 2 cm


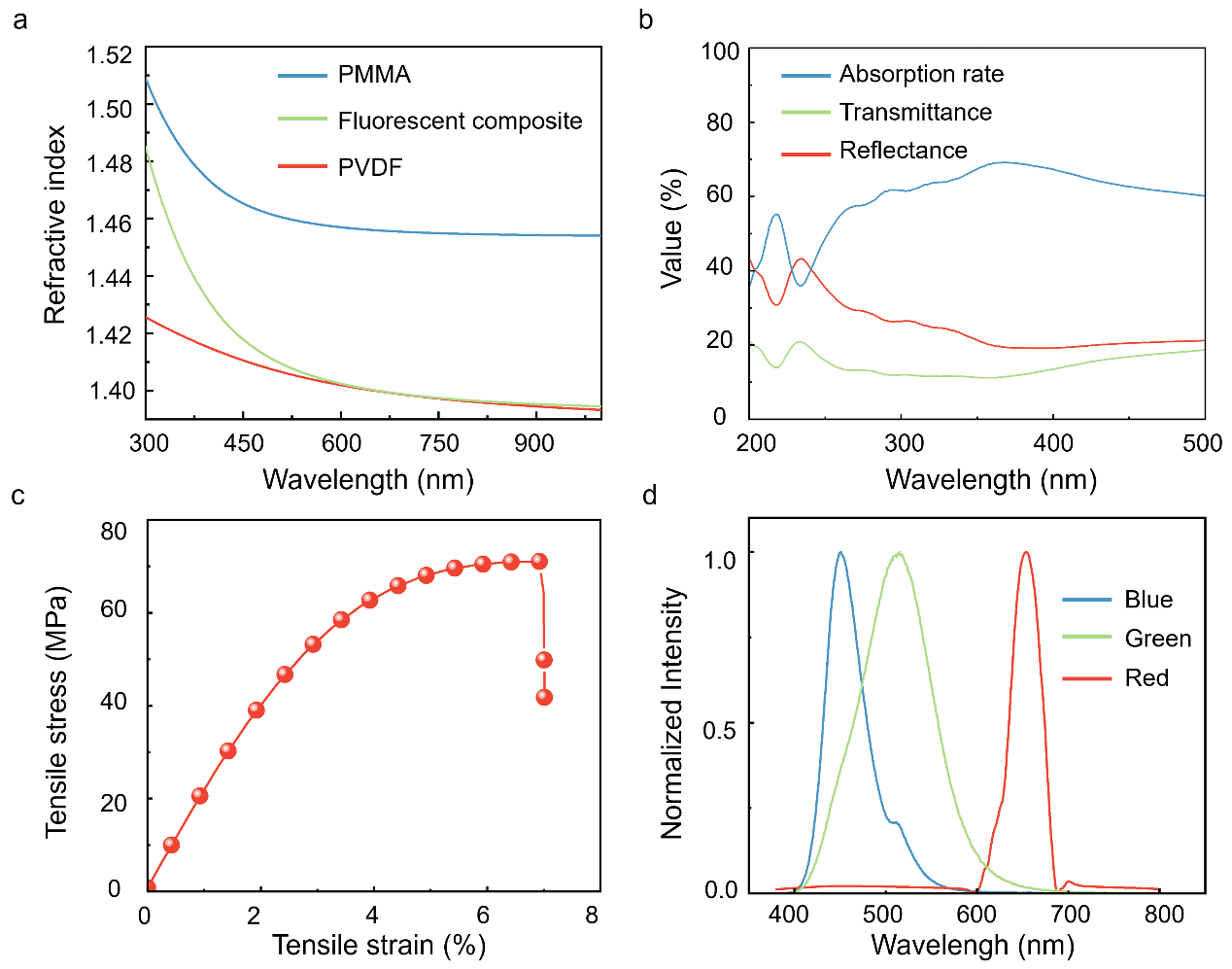


**Fig. S2 |** **a** Relative refractive index of PMMA, fluorescent composites, and PVDF. **b** Absorbance, transmittance and reflectance of fluorescent composites. **c** Stress–strain curve of photochromic fiber. **d** Fluorescence emission spectra of photochromic fiber that emit blue, green, and red light.


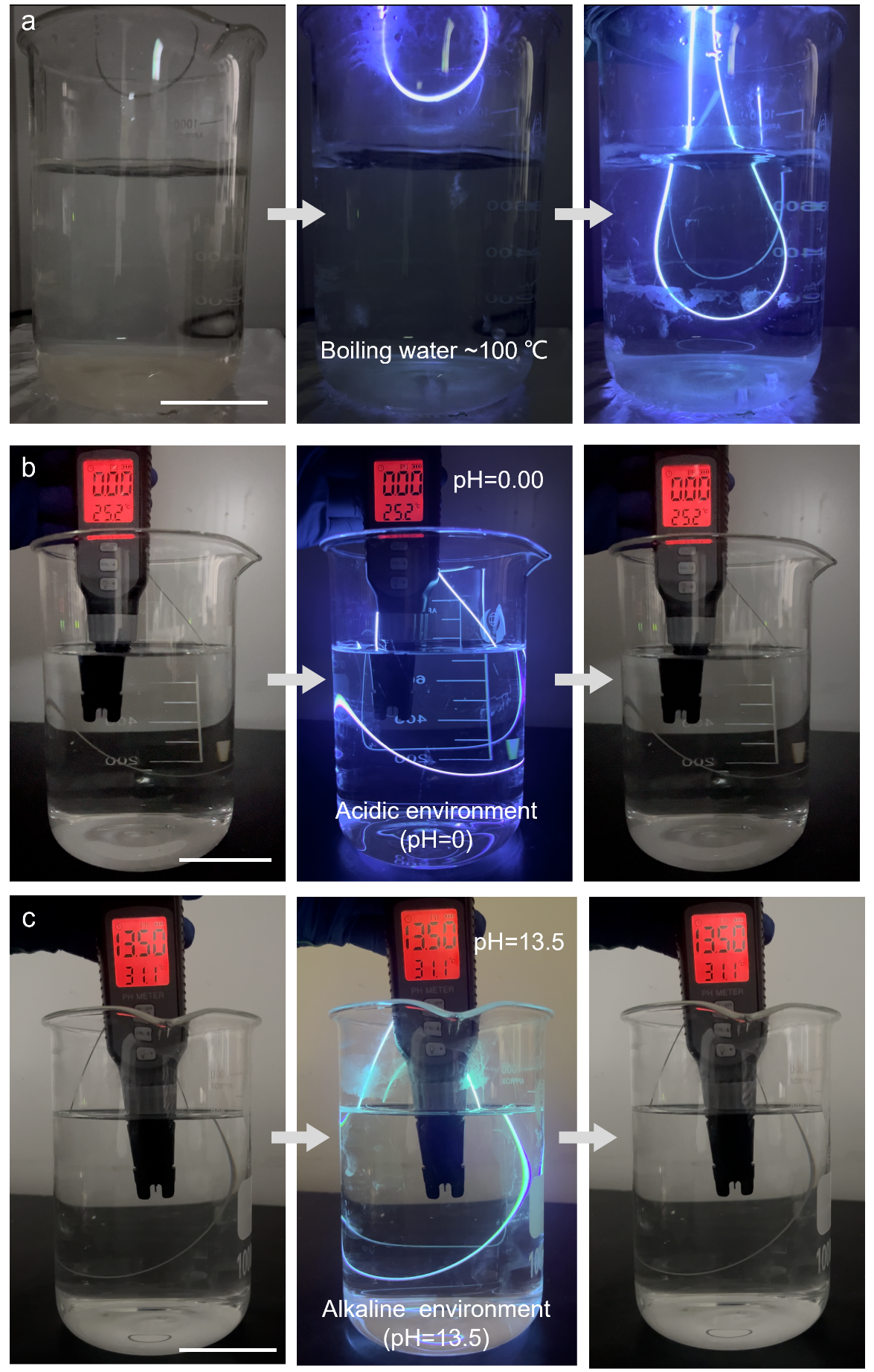


**Fig. S3 |** **a** Testing photochromic fiber in boiling water (100 ℃). **b** Testing photochromic fiber in acidic environment (pH=0). **c** Testing photochromic fiber in alkaline environment (pH=13.5). The scale bar in each case corresponds to 5 cm.


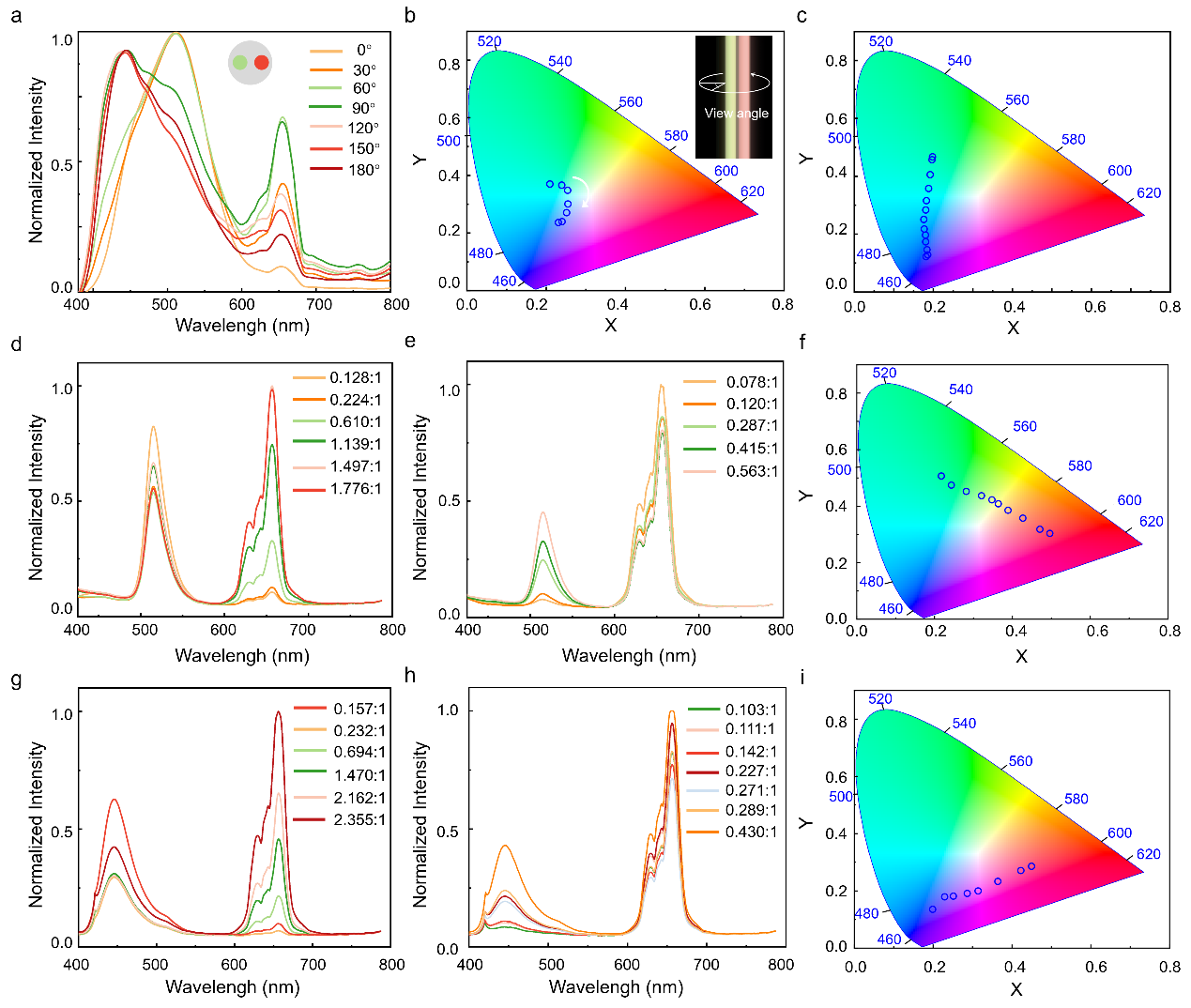


**Fig. S4 | Luminescence performance of multicolored photochromic fiber. a** The Circumferential spectra of multicolored photochromic fiber measured from 0° to 180° at 30° increments. **b** Dependence of x, y chromaticity coordinate on viewing angle. **c** x, y chromaticity coordinates controlled by adjusting the light power of different cores (green and blue). **d** Luminescence spectrum with the brightness ratios of green to red shown on the right. The power of the green core coupling light source unchanged, while the power of the red core coupling light source is adjusted. **e** Luminescence spectrum with the brightness ratios of red to blue shown on the right. The power of the red core coupling light source unchanged, while the power of the green core coupling light source is adjusted. **f** x, y chromaticity coordinates controlled by adjusting the light power of different cores (green and red). **g** Luminescence spectrum with the brightness ratios of blue to red shown on the right. The power of the blue core coupling light source unchanged, while the power of the red core coupling light source is adjusted **h** Luminescence spectrum with the brightness ratios of red to blue shown on the right. The power of the red core coupling light source unchanged, while the power of the blue core coupling light source is adjusted **i** x, y chromaticity coordinates controlled by adjusting the light power of different cores (blue and red).


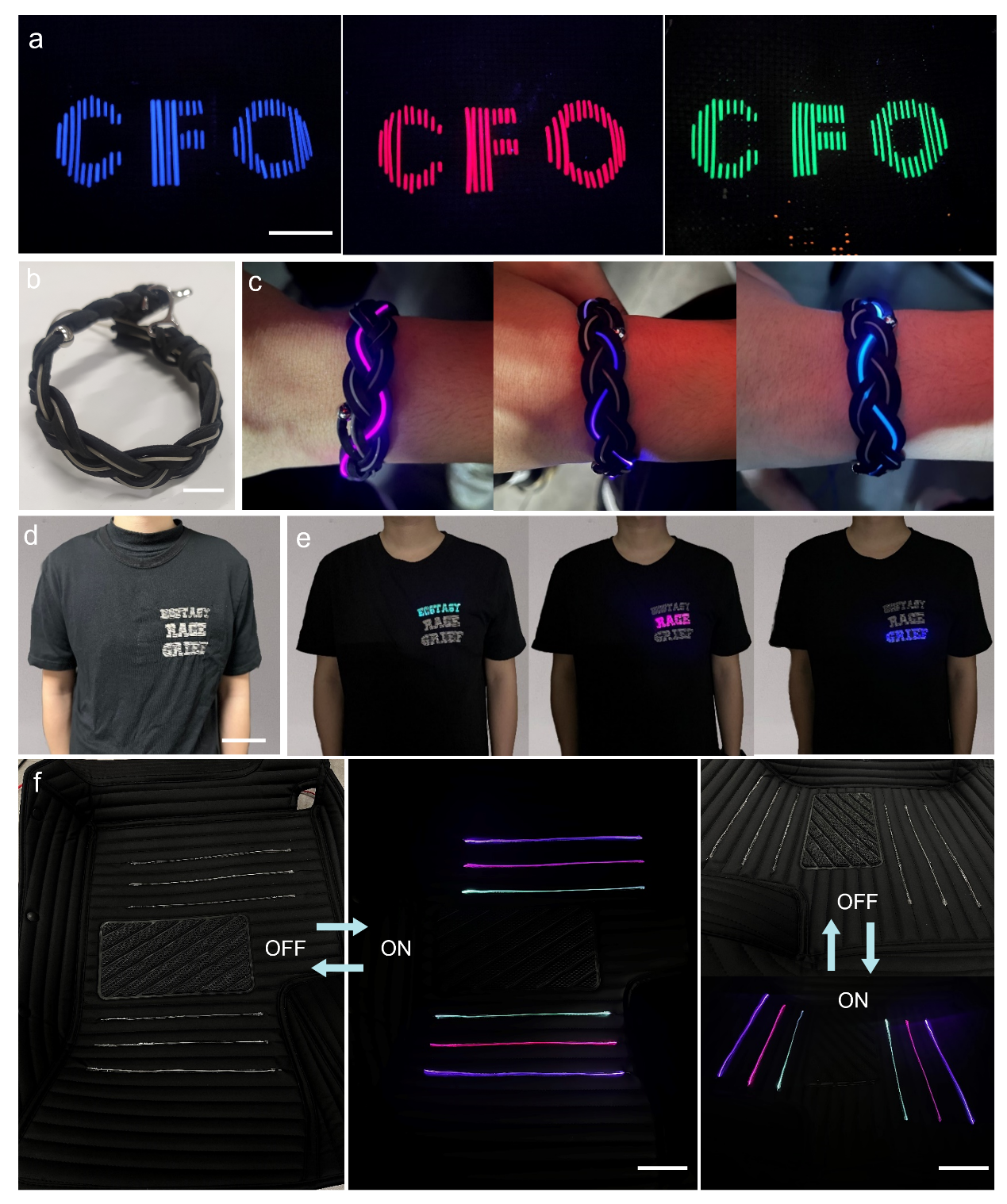


**Fig. S5 | Application scenarios of photochromic fiber. a** “CFO” pattern composed of red, green and blue photochromic fiber integrated into the cross-stitch fabric. Scale bar, 5 cm. **b** Photograph of the wearable wristband. Scale bar, 2 cm **c** Light-emitting wristband woven from red, green and blue photochromic fibers. **d** Photograph of the photochromic fiber integrated into T-shirts. Scale bar, 10 cm **e** Photograph of the corresponding emotional pattern on the T-shirts. **f** Photograph of the photochromic fiber in automotive interiors. Scale bar, 10 cm.

**Table S1** | Comparison of the current light-emitting fibers

| **Light-emitting fibers** | **Polymer optical fibers^1^** | **Light-diffusing fiber^2^** | | **Electroluminescent fiber^3^** | **Multicolored photochromic fiber (This work)** |
| --- | --- | --- | --- | --- | --- |
| **Materials** | PMMA | Silica | Hydrogel, Metal wire/ZnS phosphor | | **PMMA, PVDF/CaS** |
| **Fabrication** | Thermal drawing | Thermal drawing | Multilayer coating | | **Thermal drawing** |
| **Driving voltage** | NA | N/A | High frequency voltage over 220V | | **No more than 5V** |
| **Circumferential brightness** | Non-uniform | Uniform | Uniform | | **Uniform** |
| **Transmission direction brightness** | Decrease | Decrease | Uniform | | **Uniform** |
| **Single fiber with multicolor** | N/A | N/A | N/A | | **Trichromatic combination** |
| **Washing stability** | N/A | N/A | N/A | | **Stable** |
| **Working in extreme environments** | N/A | N/A | N/A | | **Work in 100 ℃ boiling water and strong acidic/alkaline environments** |

**Supplementary References**

1 Tan, J. Photonic patterns: fashion cutting with illuminating polymeric optical fibre (POF) textiles, *The Second International Conference for Creative Pattern Cutting, University of Huddersfield, UK.*24-25 (2016).

2 Logunov, S. et al*.* Light diffusing optical fiber for Illumination, *Solid-State and Organic Lighting.* Optica Publishing Group, DT3E. 4 (2013).

3 Yang, C. et al*.* Ionotronic luminescent fibers, fabrics, and other configurations. *Advanced. Materials.* **32**, 2005545 (2020).
